# Research on the evolution of cross-platform online public opinion for public health emergencies considering stakeholders

**DOI:** 10.1371/journal.pone.0304877

**Published:** 2024-06-25

**Authors:** Yan Shen, Zhou Luo, Xinping Song, Chunhua Liu

**Affiliations:** 1 School of Management, Jiangsu University, Zhenjiang, China; 2 School of Computer Science and Communication Engineering, Jiangsu University, Zhenjiang, China; Victoria University, AUSTRALIA

## Abstract

**Objective:**

To explore the different processes of the themes and emotional evolution of various stakeholders in the network public opinion of sudden public health emergencies at different stages of the public opinion evolution lifecycle.

**Methods:**

This paper proposes a cross-platform analysis method for online public opinion during the public health emergencies based on stakeholders. Firstly, data from multiple platforms are collected and integrated. Secondly, stakeholders are categorized and the stages of public opinion evolution are determined based on stakeholder theory and lifecycle theory. Finally, the Latent Dirichlet Allocation (LDA)+Word2vec model and Convolutional Neural Network (CNN) model are used to analyze the themes and emotional evolution of stakeholders during different stages of public opinion evolution.

**Results:**

There are differences in the evolution patterns of different types of stakeholders. The evolution process of stakeholders’ focus points exhibits a two-stage transition from concentration to divergence. The focus points of stakeholders are closely associated with their respective social domains. The emotions of the public undergo a three-stage process of positive-negative-positive change.

**Conclusions:**

This study can provide a reference for the government to have a more comprehensive understanding of the development trend of public opinion and reduce the negative impact of public opinion.

## Introduction

With the development of the mobile internet and the popularity of smart devices, the mechanism of information acquisition and transmission has changed. This change not only enables the public to stay informed about public health emergencies occurring at home and abroad, but also shifts traditional social opinion from offline to online, evolving into cyber-opinion with online platforms as the primary focus. Since the development of internet technology, cyberspace has permeated all aspects of people’s lives. People increasingly share their lives, views, and opinions on certain matters through micro-content on the internet, fostering communication and interaction with others. The willingness of individuals to express themselves on the internet has grown stronger over time, facilitated by the convenient technology and channels provided by internet advancements. According to the 52nd Statistical Report on Internet Development in China released by the China Internet Network Information Center (CNNIC), as of June 2023, China had 1.079 billion internet users, with an internet penetration rate of 76.4%. Among them, the user base of short video platforms reached 1.026 billion, marking an increase of 14.54 million compared to December 2022. Additionally, the user base of online news platforms reached 781 million, accounting for 72.4% of overall internet users [1]. Furthermore, according to an industry report by Weibo, the monthly active users of Weibo reached 599 million, with a net increase of 17 million year-on-year. The daily active users amounted to 258 million, with a net increase of 5 million year-on-year. Currently, the cross-platform social media ecosystem, including “two microblogs and one end”–Weibo, WeChat, news clients, Douyin, and others, has become the primary outlet for people to express their opinions and emotions. Consequently, when a public health event occurs, relevant public opinion information swiftly spreads across these cross-platform social media networks, resulting in a rapid outbreak of online public opinion.

Public health emergencies exhibit characteristics such as poor predictability, ferocity, wide reach, and uncertainty regarding future development. If these events are left to ferment without intervention, they are highly likely to cause significant repercussions on the internet, eventually giving rise to online public opinion that brings health threats and panic to people. To address the occurrence of public health emergencies and mitigate their negative impact on society, the government and scholars have shown great concern for the evolution process and characteristics of online public opinion surrounding such emergencies. In light of this, this study collects public opinion data on public health emergencies from various platforms. Drawing on stakeholder theory and life cycle theory, it employs LDA+Word2vec thematic analysis and CNN sentiment analysis to investigate the differentiated evolution process of themes and sentiments among stakeholders involved in cross-platform public health emergencies during different stages of the public opinion evolution life cycle. The study aims to provide valuable insights for the government to gain a more comprehensive understanding of the development of online public opinion on public health emergencies.

## Literature review

### Research on theme mining of online public opinion on public health emergencies

In the study on topic mining of online public opinion regarding public health emergencies, Farhadloo et al. conducted theme mining of Zika virus text data on Twitter. They compared and analyzed online topic discussions with the results of offline questionnaires, demonstrating the possibility of analyzing people’s views and attitudes towards public health emergencies using online public opinion data [2]. Wang et al. utilized the LDA model to mine public opinion topics on Weibo during the COVID-19 pandemic. They determined the level of public concern for each topic by counting the number of blog posts under each topic. Additionally, they analyzed user interaction behaviors across different themes by considering likes, retweets, comments, time, and region. This enabled them to study the changes in attention among microblog users in each region during the new coronavirus outbreak [3]. Wright et al. employed structured topic modeling to extract topics from over 4,000 free-text survey responses. The results indicated the critical importance of consistent and transparent communication and messaging from the government in improving compliance with measures to contain the virus, as well as protecting mental health during health emergencies [4]. Kwok et al. crawled tweet data related to COVID-19 vaccination on Twitter. They employed the LDA model to identify three types of online public opinion themes: public attitudes towards vaccination, public advocacy for epidemic prevention and control measures, and public misunderstandings and complaints about COVID-19 [5]. Zhao et al. collected online discussion data from the Zhihu platform during the COVID-19 pandemic. They used the LDA topic identification model to conduct an evolutionary analysis in terms of content and intensity. Their aim was to explore the topics of public opinion concern on the Zhihu platform during the epidemic and the changes in the degree of concern for each topic [6].

### Studies related to sentiment analysis of online public opinion on public health emergencies

In terms of research on sentiment analysis of public opinion regarding public health emergencies, Hung et al. analyzed the high-frequency words in positive and negative text data on Twitter. They discovered that positive tweets and negative tweets underestimated the severity of COVID-19 and expressed dissatisfaction with the travel restrictions imposed due to the pandemic. By examining the distribution of sentiment in tweets across different states in the, they demonstrated that positive sentiments tended to be more prevalent in states lower infection rates [7]. Mittal et al. examined the correlation between the number of diagnoses, deaths, and cures during the COVID-19 and the sentiment of Twitter public opinion, and the results showed a significant positive correlation between the number of cures and the number of tweets with positive sentiment [8]. Mou et al. developed a method for sentiment analysis of online public opinion that integrated emoticon features. Based on this, they proposed an online public opinion trend prediction model that incorporated sentiment values. They conducted a simulation of online public opinion heat during a vaccine counterfeiting using the model. The results demonstrated that incorporating emotional factors into the prediction of online public opinion heat for public health emergencies significantly improved the prediction accuracy [9]. Zhuang et al. analyzed the sentiment evolution of netizens’ comments from Wuhan and other regions on the Weibo platform. They aimed to identify differences in sentiment evolution between these two groups [10]. Bian et al. analyzed the expression public emotions in social media during major public health emergencies from a global. They focused on Sina Weibo public opinion and investigated emotional evolution patterns, thematic perspectives, and geographical diffusion, revealing several patterns of emotional evolution [11].

### Cross-platform internet public opinion related research

Yang et al. utilized scale-free networks to simulate the cross-platform social network environment. They analyzed the effects of individual factors, external friend environment, and external platform environment on user state changes based on the SEIR model. The aim was to explore the dissemination patterns of public opinion in cross-platform social networks. Additionally, they constructed a public opinion dissemination model based on cross-platform social networks and conducted simulation analysis on the model using MATLAB [12]. Zhang et al. proposed a method of public opinion sentiment analysis based on word frequency analysis and the LDA model. This method was applied to address public opinion issues that arise during the process of university epidemic control. They verified the method using cross-media public opinion examples related to university epidemic control. The objective was to provide a theoretical basis and technical support for the government to effectively manage public opinion [13]. An et al. used Sina Weibo and Douyin as data sources. They employed the ELECTRA and REDP methods for entity extraction and relationship extraction respectively on opinion text information. Subsequently, they constructed network opinion knowledge graphs for Weibo and Douyin separately. The knowledge graphs of each network were compared and analyzed [14]. Chen et al. developed a network opinion propagation model for negative corporate events based on the classical SIR propagation model. Their aim was to understand the propagation patterns of negative corporate events in the cross-platform “two microblogs and one end”. They constructed a cross-platform network environment for the “two microblogs and one end” based on a scale-free network and analyzed the main parameters. The results demonstrated that the constructed network public opinion dissemination model accurately described the process of network public opinion dissemination in a real environment [15].

The existing literature undoubtedly provides valuable insights and merits acknowledgment. However, this paper aims to complement and enhance the current understanding of online public opinion on public health emergencies in the following aspects: Firstly, regarding the research subject, there is a scarcity of scholars who have explored online public opinion from the perspective of stakeholders. This study addresses this gap by integrating stakeholders into the analysis of online public opinion during public health emergencies. It categorizes stakeholders and investigates the themes of concern and the emotional evolution of various stakeholders at each stage of public opinion evolution. Secondly, with respect to platforms, most current studies focus solely on analyzing the evolution of online public opinion on public health emergencies within a single platform. While some studies have examined the propagation patterns of cross-platform online public opinion events from a propagation perspective, these studies often rely on simulations rather than actual data analysis. In contrast, this study takes into account public opinion data from multiple platforms. It integrates and analyzes the collected data from various platforms to provide a comprehensive reference for the government and relevant management departments in understanding the evolving patterns and development trends of public opinion.

## Methods

**Fig 1:**
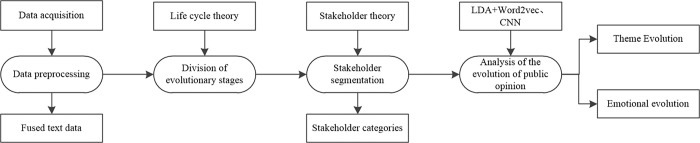
Research methods.

The public opinion evolution model is divided into theme evolution and emotion evolution. For the study of public health emergencies, this paper presents the research methodology as depicted in Fig 1. Firstly, a crawler tool is utilized to independently collect data from each platform, followed by their consolidation. Secondly, the stages of public opinion evolution are determined based on the life cycle theory. Thirdly, stakeholders are categorized and identified using the stakeholder theory, enabling the identification of specific stakeholder categories involved in the evolution of online public opinion on public health emergencies. Lastly, the CNN convolutional neural network model is employed to classify the sentiment polarity of text, while the LDA+Word2vec model is utilized to analyze the theme evolution of online public opinion.

Our data is in a file called "S1 File" in the Support Information. We promise that our data collection and analysis methods are in accordance with the terms and conditions of the PLOS ONE Journal Data Source. In addition, we store our code in a file called "S2 File" in the Support Information.

### Life cycle division of online public opinion on public health emergencies

As early as 1986, STEVEN (1986) proposed a division of emergency spread into four stages in the field of crisis management: the incubation period, outbreak period, spreading period, and recovery period [16]. Currently, there are multiple bases and methods of division in academia, such as the three-stage theory, four-stage theory, five-stage theory, and six-stage theory of online public opinion. Based on the characteristics of public health emergencies in information dissemination and STEVEN’s classical life cycle theory, this study identifies the inflection point of information volume regarding public health emergencies. Additionally, it examines the landmark events at key time points to divide the life cycle of public health emergencies into five stages: the germination period, outbreak period, spreading period, decline period, and recovery period.

Germination period. In the germination period, although the cause of the event already exists, but due to the characteristics of the event is not obvious, only a small number of users pay attention to the event. The media and government departments do not pay much attention to the event. The amount of information related to the event is relatively small, and is in the state of slow increase.

Germination period: During this phase, although the cause of the event already exists, it remains relatively inconspicuous, with only a small number of users paying attention to it. Media and government departments do not allocate significant attention to the event. The volume of information related to the event is relatively small and increases slowly.

Outbreak period: As the event triggers continue to accumulate, the event’s influence and scope of impact expand rapidly. The event begins spreading across major social media platforms with significant momentum. During this stage, more users become interested in the incident, leading to a rapid increase in the amount of information related to the event until it reaches its peak.

Spreading period: In this stage, the incident continues to spread, and more users engage in discussions about it. However, relevant government interventions begin to control the situation, causing some uninvolved individuals to lose interest. As a result, the amount of information related to the event shows a decreasing trend. However, the management of public health emergencies tends to involve repetitive actions, leading to longer durations of the spreading period. Consequently, the amount of information about the event fluctuates and decreases.

Decline period: During the decline period, the intensity of the event starts diminishing, and social order gradually restores. Only a small portion of the population continues to pay attention to the event, with the public’s focus shifting to other matters. Although the amount of information about the event fluctuates, it generally decreases.

Recovery period: In the recovery period, the incident has essentially come to an end, and the number of individuals participating in discussions about the event further diminishes. Only a very small number of users contribute a small amount of information, resulting in minimal fluctuations in the amount of information, which remains stable.

### Identification and delineation of stakeholders

#### Composition of stakeholders in each platform of public health emergencies

Due to factors such as platform positioning and user habits, the categories of stakeholders and their proportions differ across different platforms in the context of public health emergencies. Therefore, analyzing data solely from a single platform cannot fully capture the evolution of public opinion events. Integrating data from various platforms allows for a more comprehensive understanding of the thematic and emotional evolution of public opinion events, providing valuable guidance for the government in managing public opinion.

**Fig 2:**
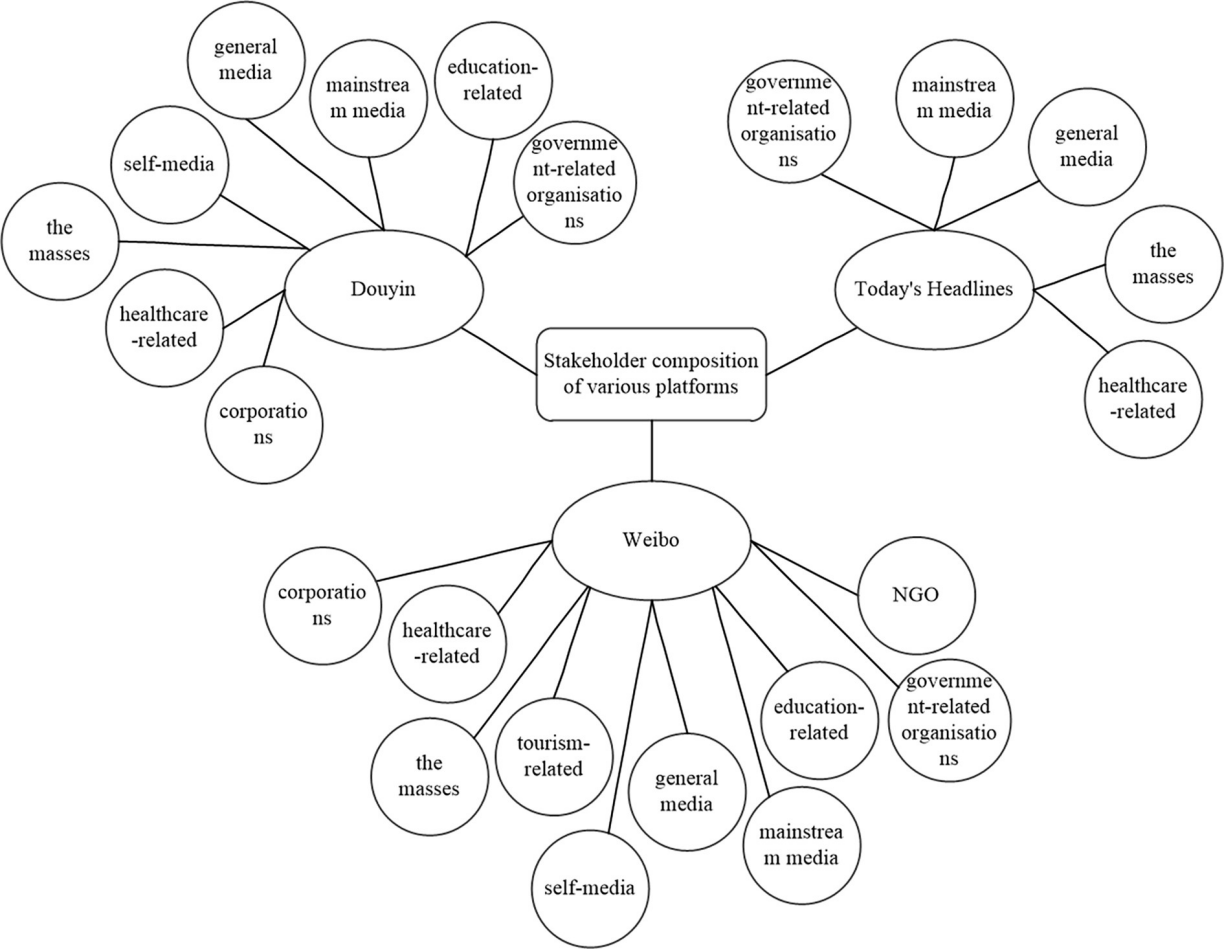
The stakeholder composition of various platforms.

Public health emergencies involve multiple stakeholders, including the government, media, healthcare sector, and enterprises, among others. In accordance with stakeholder theory and relevant literature [17, 18], and based on the data collected from each platform, the types of stakeholders involved in public opinion events on each platform are sorted and analyzed, as illustrated in Fig 2. For the purpose of this study, data from three platforms, namely Weibo, Douyin, and Today’s Headlines, are selected, considering data availability. As shown in Fig 2, Weibo, one of the largest social media platforms, encompasses the largest number of stakeholders, followed by Douyin. Today’s Headlines, being a news and information platform, includes a group of stakeholders primarily from the media category. Additionally, government-related organizations, healthcare-related stakeholders, and other stakeholders express their views on these platforms.

#### Stakeholder division

The stakeholders involved in this study are in the following 10 categories: government-related organizations, education-related, mainstream media, general media, self-media, tourism-related, the masses, healthcare-related, corporations, and non-governmental organizations (NGO).

This study applies a method that utilizes authentication information and keywords in user profiles to classify and identify users as stakeholders. Users are classified by matching their authentication level and authentication information. In cases where authentication information is unavailable, the user’s username and keywords in their profile are used to determine their specific stakeholder category. The specific classification criteria are presented in Table 1. In this study, we chose two indicators, the average of postings and the average of followers, to categorize mainstream media and general media. We first categorized all media category stakeholders. Then, we calculated the average value of post volume and the average value of number of fans for all media category stakeholders. Finally, we classify the media whose post volume and follower count are higher than the average value as mainstream media, and the rest as general media.

**Table 1:** Classification criteria for various stakeholders.

stakeholder	Criteria for classification	
	Certification Information	Introduction keywords
government-related organizations	Health Commission, Party Committee, People’s Government, and Center for Disease Control and Prevention	government, party committees, courts, procuratorates
education-related	schools, school boards, libraries, knowledge and science bloggers	schools, colleges, education department, popularization of knowledge
mainstream media	media, newspapers, radio, magazines	media, newspapers, radio, magazines
general media	media, newspapers, radio, magazines	media, newspapers, radio, magazines
self-media	author, video information blogger, entertainment blogger	writer, blogger, commentator
tourism-related	tourism organizations	tourism, tourism board, Culture and Tourism
healthcare-related	hospitals, doctors, health service center	hospitals, doctors, physicians
corporations	companies, associations	companies, enterprises, associations
non-governmental organizations (NGO)	civil society organizations such as the Disabled Persons’ Federation, Women’s Federation, Confederation, Trade Unions, etc.	disabled persons’ federations, women’s federations, trade unions, confederations
the masses	the masses	-

### Theme extraction

#### LDA thematic modeling

With the advancement of text mining technology, various topic mining techniques have emerged, including TF-IDF, TextRank, and other algorithmic models. While these algorithms can extract topic features from text data, they often overlook the semantic relationships within the text and fail to effectively capture the underlying connections between topic words. Therefore, this paper adopts the LDA to mine textual topics, taking into account the contextual semantics and aiming to capture the inherent relationships between topics.

**Fig 3:**
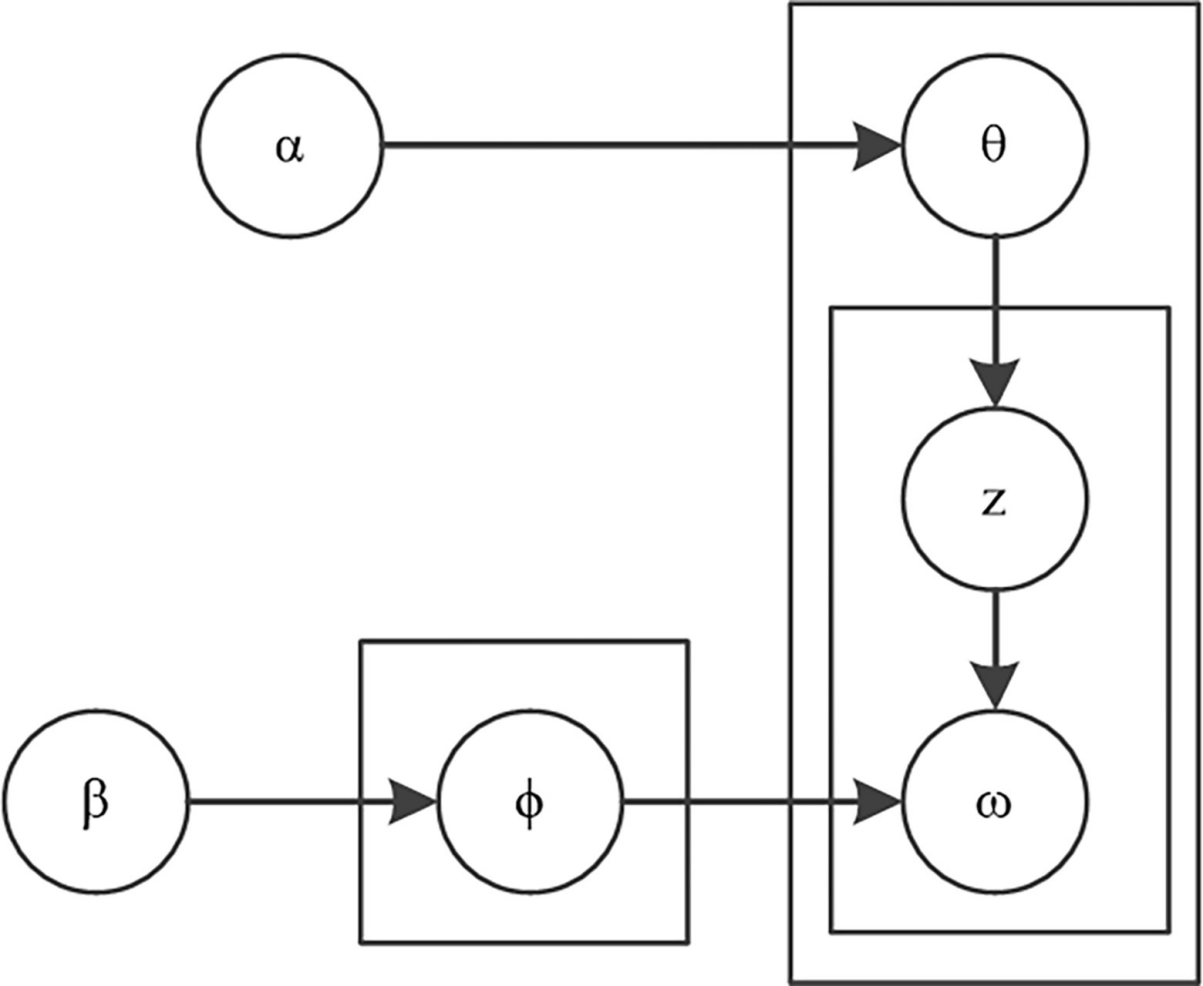
LDA model structure diagram.

LDA (Latent Dirichlet Allocation) is an unsupervised clustering algorithm based on Bayesian ideas proposed by Blei et al [19], which is widely used in the scenarios of text clustering, text analysis, text keywords, etc. The structure of the model is shown in Fig 3, where *α* and *β* are the distribution hyperparameters of the topic distribution *θ* and the topic word distribution *ϕ*, respectively [20]. *z* and *ω* are the final topics and topic words generated by the model, respectively.

The specific generation process of themes and theme words is as follows: ① Generate the theme distribution of the document *θ*_*i*_ by sampling from the Dirichlet distribution *α*; ② Generate the theme *z*_*i*,*j*_ of the *j*th word of the document *i* by sampling from the polynomial distribution of the theme *θ*_*i*_; ③ Generate the distribution of the words $\phi_{z_{i,j}}$ corresponding to the theme *z*_*i*,*j*_ by sampling from the Dirichlet distribution *β*; ④ Generate the words *ω*_*i*,*j*_ by sampling from the polynomial distribution of the words $\phi_{z_{i,j}}$, and the joint distribution is shown in Eq (1).


$$P\left(\omega_{i},z_{i},\theta_{i},\phi |\alpha ,\beta \right)=\prod_{j=1}^{N}p(\theta_{i}|\alpha )p(z_{i,j}|\theta_{i})p(\phi |\beta )p(\omega_{i,j}|\phi_{z_{i,j}})$$ (1)

#### Word2vec word vector modeling

The Word2vec model is currently the dominant distributed representation model for words [21]. Word2vec consists of two models, CBOW and Skip-Gram. The former predicts the center word by context words, while the latter predicts the context words given the center word. The CBOW model has higher computational accuracy than the Skip-Gram model, and is chosen for this paper.

The Word2vec model solves the problem of large and sparse vector space to a certain extent, while preserving the relationship between words. Additionally, Word2vec utilizes the Negative Sampling method, which accelerates the learning speed and reduces the computational requirements, thus achieving efficient learning. Therefore, this study adopts the approach of combining LDA with Word2vec for text topic analysis.

### Emotional analysis

Sentiment classification is the process of analyzing, processing and summarizing text with sentiment. Traditional sentiment classification is mainly based on lexicon or feature engineering, which requires tedious manual feature design and a priori knowledge, and the understanding stays at a shallow level with poor scalability. The proposal of deep learning solves the above problems. Currently, deep learning technology has been widely used in various fields [22, 23]. Deep learning-based sentiment propensity classification models are able to provide end-to-end semantic understanding of the input text and judge the sentiment propensity based on the semantic representation. The convolutional neural network model can automatically learn the features in the input data without manually designing the feature extractor, and the convolution operation can capture the local features of the input data, so that the model can better understand the structure and content of the data, and extract the features through the convolutional layer and the pooling layer, which can efficiently extract the local features in the text, so as to achieve the purpose of judging the emotion of the utterance. In contrast, logistic regression models can only perform linear transformations, making it difficult to capture complex semantic relationships in text. In addition, there have been studies demonstrating the superiority of CNN models in sentiment classification tasks compared to logistic regression models. Smith et al. compared the logistic regression model and CNN model on the same sentiment dataset in terms of accuracy, recall and other metrics. The experimental results show that the CNN model has better performance in the sentiment analysis task compared to the logistic regression model. The CNN model achieves higher accuracy in the sentiment classification task [24]. Dhammi et al. investigated the application of Convolutional Neural Networks (CNN) and Logistic Regression models in task of sentiment analysis on Twitter. The experimental results demonstrated that the CNN model significantly outperformed the logistic regression model in the task of text sentiment classification [25]. Kaur et al. conducted a comparative analysis of logistic regression and convolutional neural network (CNN) models for sentiment analysis of financial news. The study also investigated the impact of different feature representations on the performance of the models and validated the advantages of CNN models in text sentiment classification tasks [26]. Based on this, this study adopts the convolutional neural network model CNN based on the large language model to classify the emotional tendency of text data.

**Fig 4:**
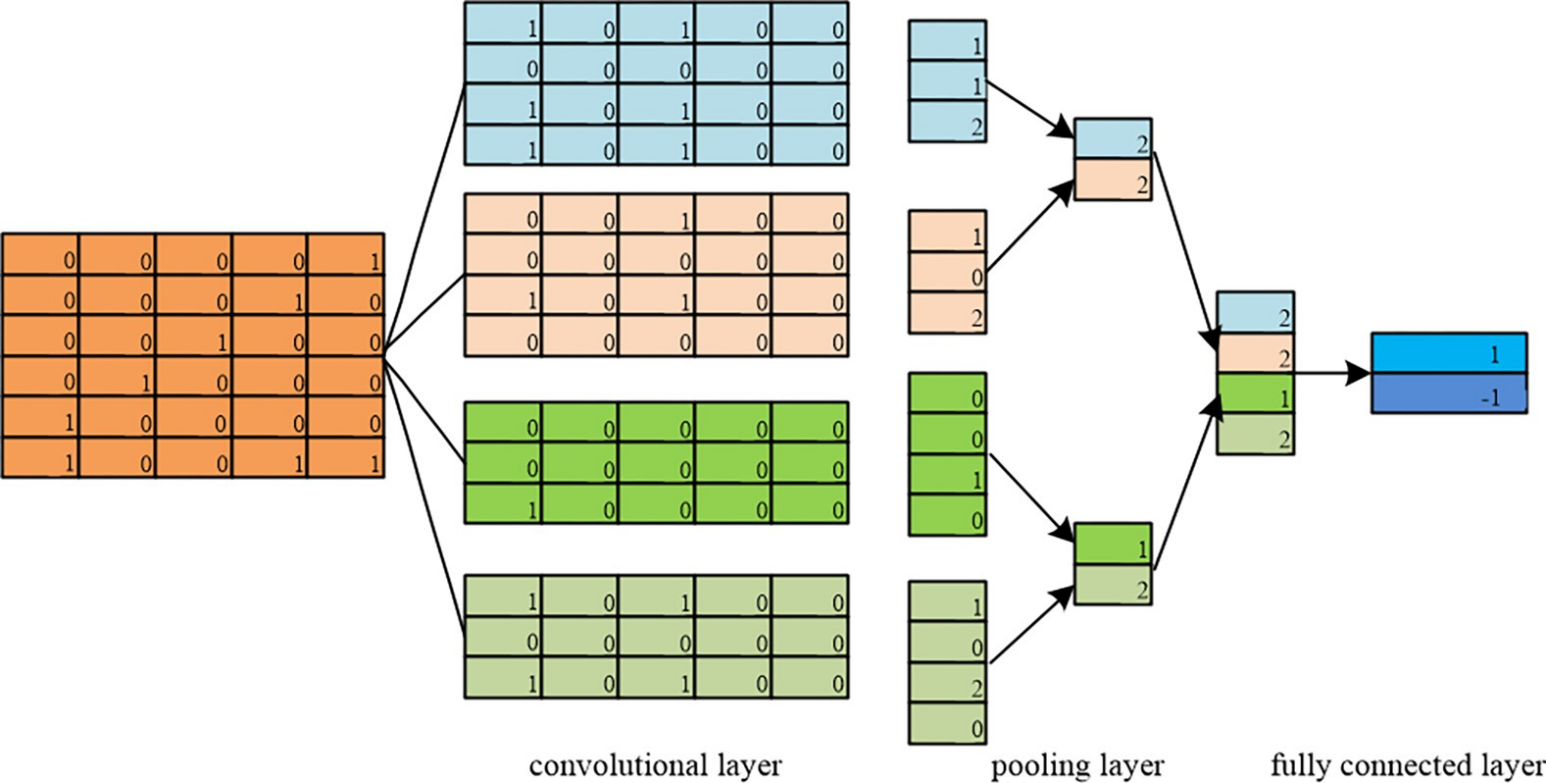
Convolutional neural network sentiment classification structure diagram.

In general, the CNN model is mainly composed of four parts: the input layer, convolutional layer, pooling layer, and fully connected layer. Fig 4 illustrates the structure of the Convolutional Neural Network (CNN) for sentiment classification.

For a sentence of length *n*, assuming that *x*_*i*_ is the *k*-dimensional vector of the *i*th word in the sentence, this sentence of length *n* can be represented in the input layer as:

$$x_{1:n}=x_{1}⊕x_{2}⊕\cdots ⊕x_{n}$$ (2)

where ⊕ is the concatenation operation.

In the convolutional layer, assume that the input feature matrix is *w*∈*R*^*n*×*k*^, its sub-matrix is *w*_*i*_∈*R*^*h*×*k*^ and the convolution kernel is $x_{i:i+h-1}\in R^{k\times h}$, then the output after convolutional computation is:

$$c_{i}=f(w_{i}\cdot x_{i:i+h-1}+b)$$ (3)

where *f* is the activation function and *b* is the bias top.

In order to avoid the overfitting phenomenon, the data features output from the convolutional layer need to be compressed and downgraded in the pooling layer. By reducing the dimensionality of the word vectors, the model computation can be greatly reduced, thus avoiding the overfitting problem efficiently. Finally, in the fully connected layer, a classifier is used to normalize the resulting feature vectors to obtain the score of the current text, and the corresponding term of its resulting maximum probability value is the corresponding sentiment polarity of the text.

The specific parameters of the CNN model used in this paper are shown in Table 2.

**Table 2:** Model parameter setting.

parameters	value
num filter	256
kernel size	[2, 3, 4]
hidden size	128
word_emb_dim	128
learning rate	0.01
max_epoch	10
batch_size	128
max_grad_norm	5.0

## Results

### Data acquisition and pre-processing

Taking the "Norovirus" event as an example, the crawler tool is used to collect data from Weibo, Douyin, and Today’s Headlines with "Norovirus" as the search keyword. The collection fields include: publisher id, publisher name, posting time, posting content, publisher authentication information, publisher profile information, number of followers, etc. The collection time period is set from 1 February 2023 to 15 March 2023 based on the Baidu index curve of the "Norovirus" event. The text data collected from the three platforms are fused, the specific operations are: ① pattern matching; ② entity alignment; ③ data fusion. The fused text data are subjected to data cleaning with the following rules: ① Delete duplicate contents posted by the same user and keep only one; ② Remove links, @ other users, and special symbols in the text. Finally, 29277 cleaned data were obtained.

Table 3 shows some examples of sample data.

**Table 3:** Sample data.

publisher id	publisher name	posting time	posting content	publisher authentication information	publisher profile information	number of followers
1743321831	Hangzhou Normal University	2023/2/16	What are the symptoms of Norovirus Gastroenteritis…	Ranked among the Top 100 Chinese Universities in the World University Rankings	Hangzhou Normal University official microblogging	225212
3546332963	Qianjiang Evening News	2023/2/3	Norovirus-infected diarrhea enters a period of high prevalence…	All-media tourism studio of Zhejiang Newspaper Group…	Qianjiang Evening News	17546
6554376707	Dr. Yanzu Tan	2023/2/26	Multiple CDCs issue reminder of norovirus…	doctor	Excellent medical skills and high medical ethics	1490159

### Delineation of the stages of evolution of public opinion

**Fig 5:**
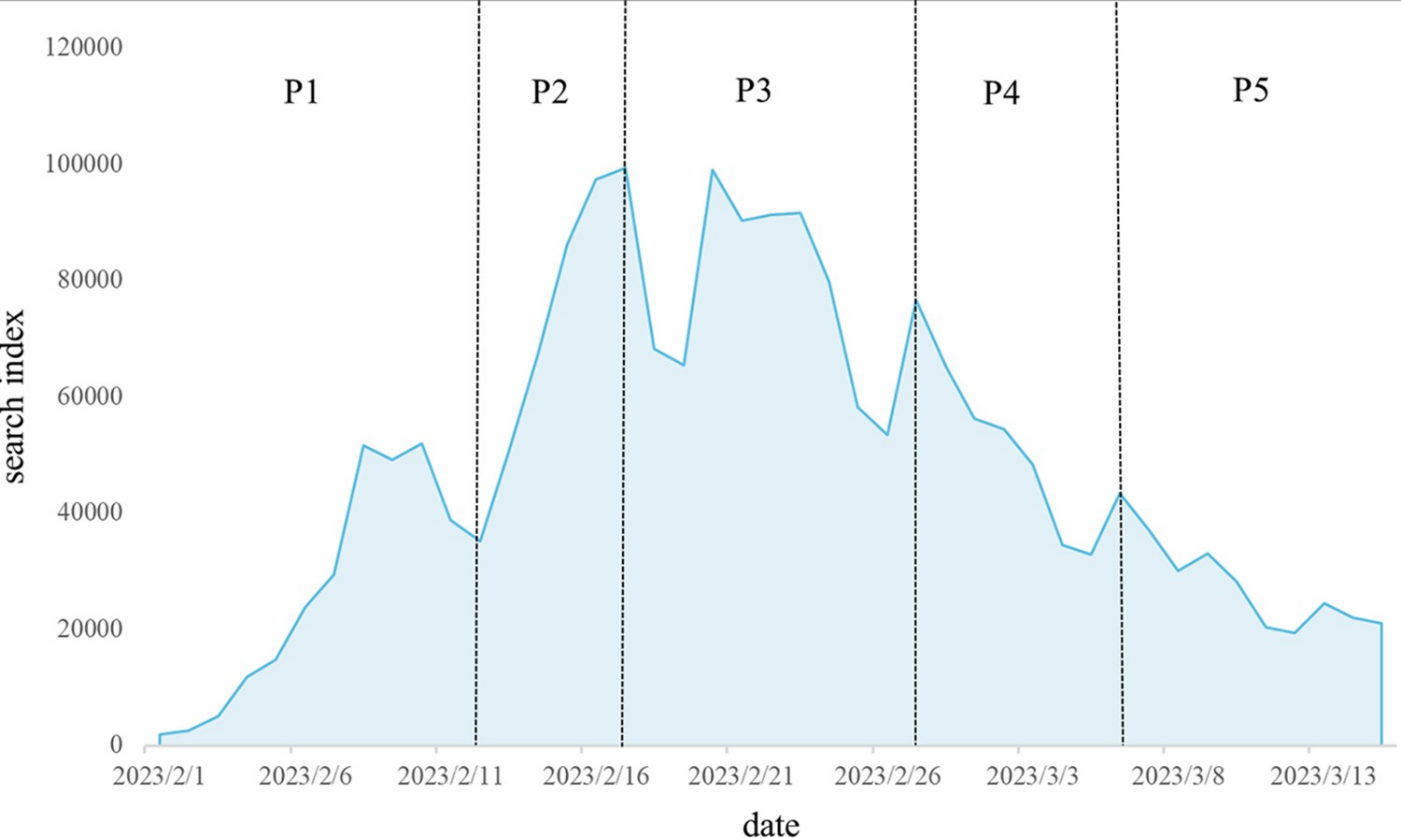
Baidu index curve of "Norovirus".

Fig 5 shows the Baidu index curve of "Norovirus", from Fig 5, we can observe several obvious inflection points, respectively, February 12, February 17, February 27, March 6, combined with the history of the release of the event, "February 10th CDC issued a reminder that norovirus has entered the high incidence period "; "On 17 February CDC released knowledge about norovirus prevention, and major media outlets scrambled to forward it"; and "On 26 February major media outlets began to report less about norovirus, and the public’s attention began to shift". According to the principle of life cycle division proposed in this study, the evolutionary cycle of the studied cases was divided into five stages: germination period P1 (1 February-12 February), outbreak period P2 (13 February-17 February), spread period P3 (18 February-27 February), decline period P4 (28 February-6 March), and recovery period P5 (7 March-15 March).

### Stakeholder theme evolution

When using LDA for topic extraction, in order to ensure the independence of topics, the indicator of topic confusion is used to determine the optimal number of topics, which is calculated as shown in Eq (4), where the denominator is the sum of all words in the test set *D* and *p*(*w*_*d*_) is the probability of occurrence of each word *w*_*d*_ in the test set.

$$Perplexity(D)=\exp\{\frac{-\sum_{d=1}^{M}\log(p(w_{d}))}{\sum_{d=1}^{M}N_{d}}\}$$ (4)

**Fig 6:**
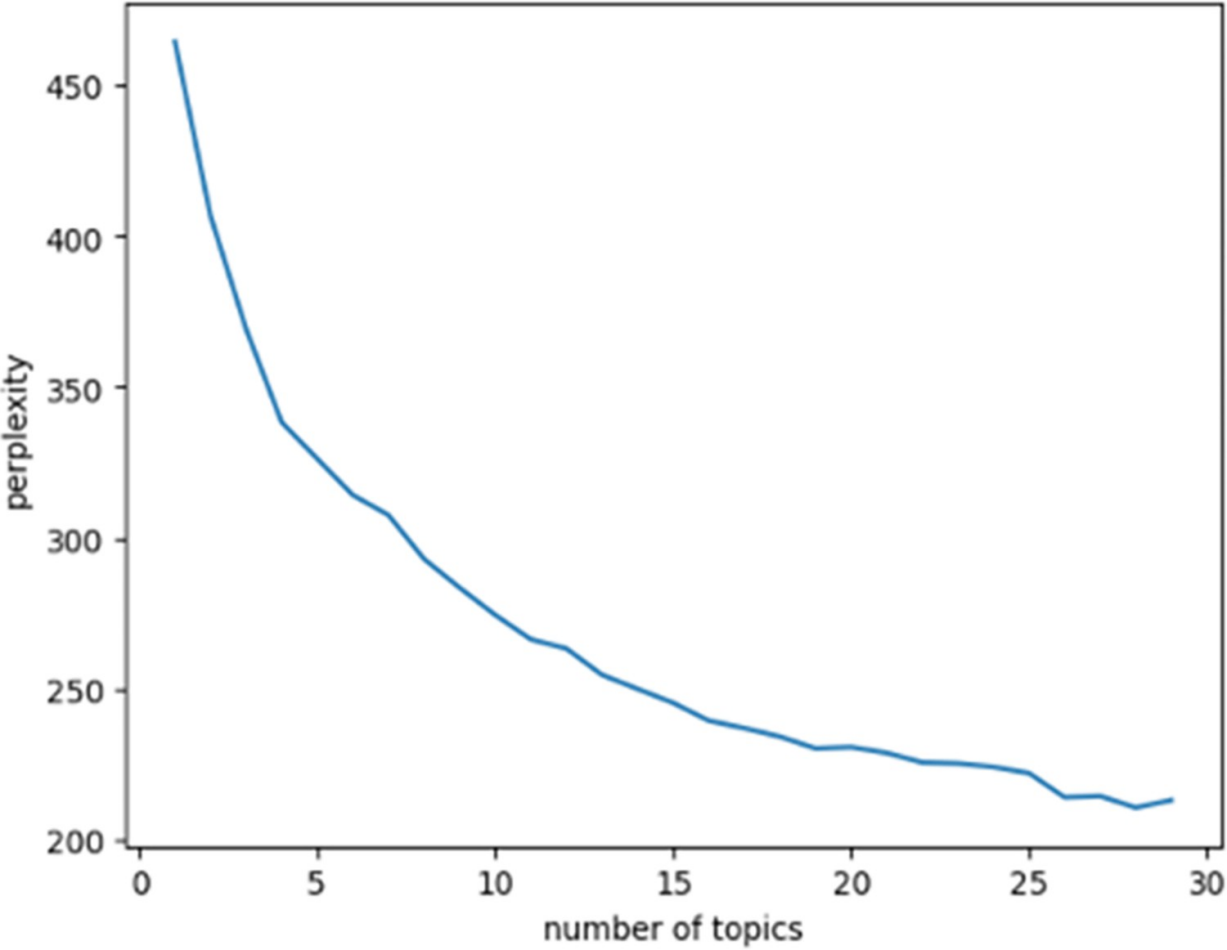
Topic perplexity curve.

We calculate the topic perplexity for different numbers of topics in the LDA model and plot the topic perplexity curve, as shown in Fig 6. We observe that when the number of topics, denoted as K, is set to 26, the topic perplexity reaches a minimum and the topic extraction results become relatively stable.

Table 4 shows the meaning of each theme and the characteristic words of each theme.

**Table 4:** Theme meaning and theme feature words.

serial number	Thematic implications	Thematic trait words
T0	Influenza virus causes pneumonia	Influenza Virus, Hospital, Influenza, Pneumonia, Expert,
T1	Sterilization for virus prevention	Sterilization, Food, Contamination, Processing, Disinfectant
T2	Causes of the high incidence of infectious diseases and routes of virus transmission	Infectious Disease, Transmission, Virus, Immunity, Pathway
T3	Expert answers to situations that require medical attention	Influenza, Situations, Experts, Infectious Diseases, Influenza Virus
T4	A number of children at the kindergarten showed symptoms of infection	Children, Parents, Kindergarten, Symptoms, Teachers
T5	Popularization of knowledge on virus prevention	Family, Tips, CDC, Science, Symptoms
T6	CDC warns of high incidence of norovirus	CDC, Peak, Infectious, Department, Virus
T7	Precautions after infection with the virus	Rehydration, Diet, Body, Disease, Virus
T8	Guidelines for the prevention of springtime infectious diseases	Infectious Diseases, Epidemic, Influenza, Guidelines, Season
T9	Doctors’ advice for the flu	Mask, oseltamivir, advice, vaccine, doctor
T10	Infectious Disease Hot Questions and Answers	Influenza, Symptoms, Infectious Diseases, Advice, Situations
T11	Deployment of national medical treatment	Medical, work, National, Influenza, Deployment
T12	Detection of viruses	Virus, Tests, Nucleic Acid, Symptoms, Hospitals
T13	Norovirus enters high season in Guangdong	High incidence period, Guangdong Norovirus, Virus, Doctor, Transmission
T14	Popularization of Norovirus-related knowledge	Popular Science, Program, Medicine, Guides, Hot Topics
T15	Norovirus is vulnerable to outbreaks in closed environments, such as schools and childcare facilities	Populations, Schools, Childcare Institutions, Transmission, Environment
T16	Norovirus infection in students	Students, Primary Schools, Infections, Education Bureau, Dissemination
T17	Symptoms in infected children as well as adult patients	Patients, Symptoms, Children, Adults, Gastroenteritis
T18	CDC reminds localities to prevent and control norovirus outbreaks at the start of the school year	Schools, Students, CDC, Start of School, Influenza
T19	Multiple CDCs issue norovirus prevention and control measures	Prevention and Control, Measures, Dissemination, CDC, Disinfectant
T20	CDC reminds that it is the high season for Norovirus gastroenteritis	Gastroenteritis, Symptoms, Outbreak, Season, CDC
T21	Differences in symptoms between influenza A and norovirus	Symptoms, Influenza, Digestive tract, Muscle, soreness
T22	Sharing of Norovirus Infection	Diarrhea, Mum, Sick, Child, Hospital
T23	An explanation for why there is no norovirus vaccine on the market	Epidemic, Vaccine, Human, Situation, Virus
T24	Main symptoms of Norovirus gastroenteritis	Gastroenteritis, Main Symptoms, Symptoms, Spread, Physician
T25	CDC Issues Virus Family Prevention Tips	Symptoms, CDC, Soap, Disinfection, Flushing

**Fig 7:**
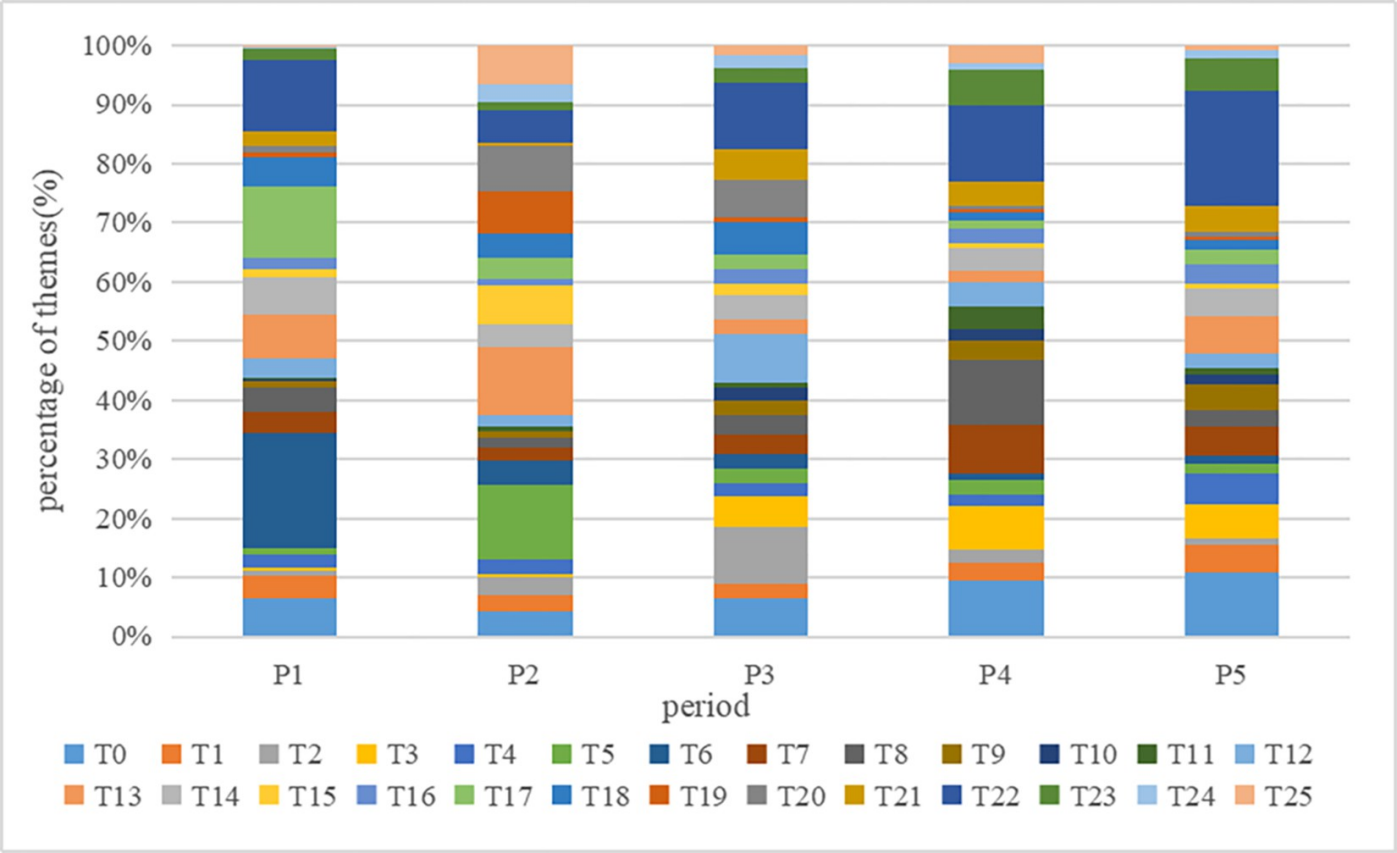
Thematic evolution accumulation map.

The number of texts for each theme in different periods is tallied, and the evolution of themes is depicted in Fig 7. Overall, the theme "sharing of norovirus infection (T22)" emerges as the most prevalent among all themes. This prominence can be attributed to the fact that during public health emergencies, the general public constitutes a significant portion of the population. Consequently, when such events impact them, the public expresses their emotions and opinions through social media. Analyzing the distribution of theme proportions across each period, it becomes evident that certain themes dominate in specific periods while having a diminished presence in others. For instance, the theme "CDC warns of high incidence of norovirus (T6)" accounted for the highest proportion during the initial stage, indicating that governmental departments started paying attention to the event at its early onset.

### Stakeholder concerns

**Fig 8:**
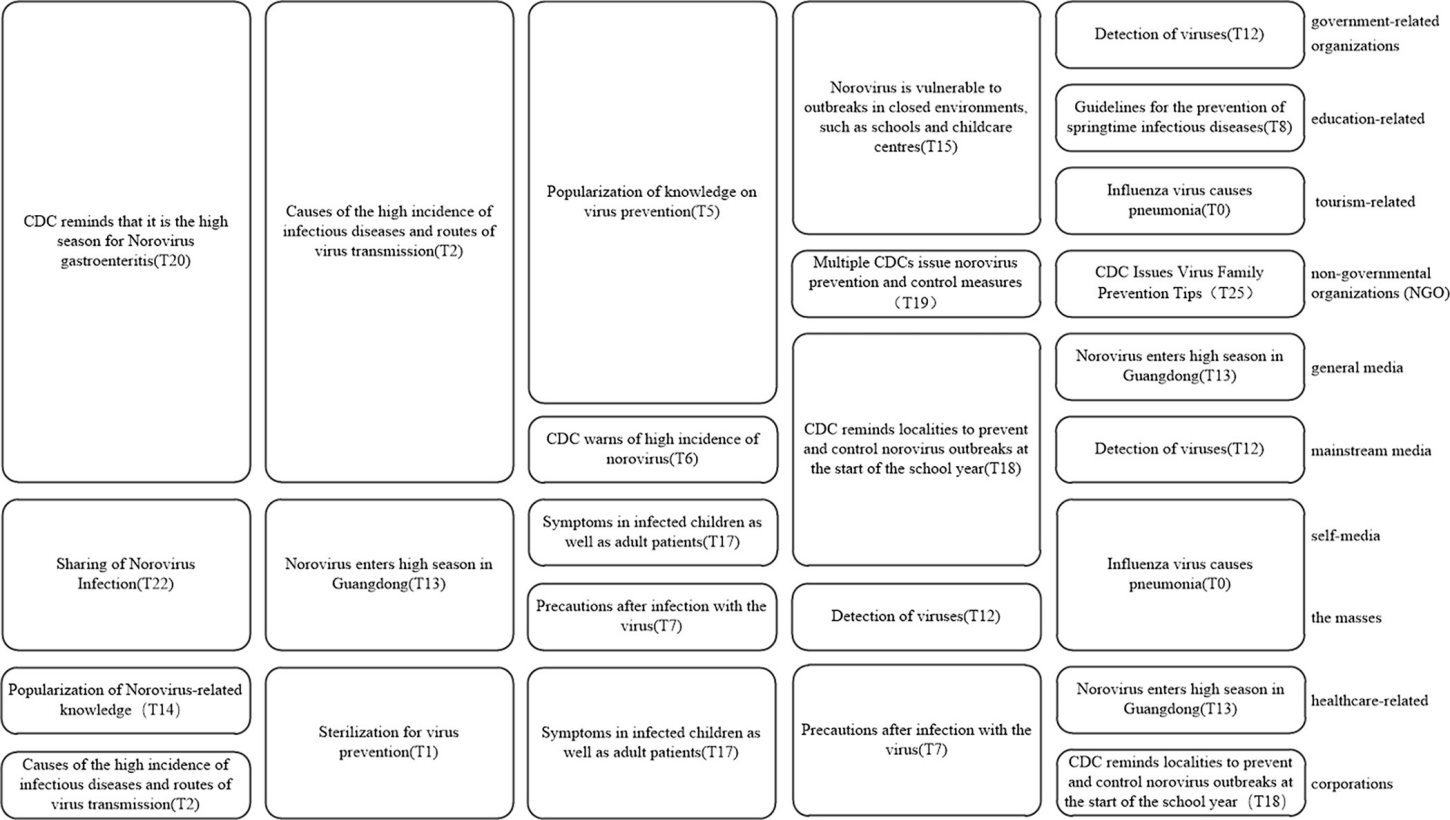
Comparison of concerns of different stakeholders.

To further elucidate the variations in topics of concern among stakeholders during the evolution of public opinion, we tally the top five topics of interest for each stakeholder group and summarize their concerns, as illustrated in Fig 8. Fig 8 represents the five topics that different stakeholders are most concerned about during the evolution of public opinion. In Fig 8, the text in the box represents the topics that stakeholders are concerned about. Each row represents the five topics that the stakeholders in that category are most concerned about.

From Fig 8, it can be visualized that the themes of concern of the three groups of stakeholders, namely government-related organizations, education-related and tourism-related, are highly overlapping, and NGO as well as the general media also have three themes that are consistent with the above three. Self-media and the masses, as well as healthcare-related and corporations, each have three consistent themes. The themes with high levels of concern were summarized and can be broadly categorized into event alerts (T20, T18, T13, T15), event causes and transmission pathways (T2), mass sharing (T22), knowledge and virus prevention (T1, T5, T7), virus testing (T12), and disease symptoms (T0, T17).

### Patterns of stakeholder opinion evolution

We tally the text distribution of topics of concern for each stakeholder during each stage of the public opinion life cycle. The topic that ranks highest in each stage for each stakeholder type is considered representative of the concerns within that period for that stakeholder group. By doing so, we obtain the distribution of concerns for each stakeholder during each stage of the life cycle, as presented in Table 5.

**Table 5:** Distribution of concerns of stakeholders at each stage.

Stakeholder categories	P1	P2	P3	P4	P5
government-related organizations	Topic6	Topic5	Topic2	Topic8	Topic0
education-related	Topic6	Topic5	Topic2	Topic8	Topic8
mainstream media	Topic17	Topic20	Topic20	Topic8	Topic4
general media	Topic6	Topic5	Topic2	Topic8	Topic21
self-media	Topic17	Topic13	Topic22	Topic23	Topic22
tourism-related	Topic18	Topic5	Topic2	Topic7	Topic0
the masses	Topic22	Topic13	Topic22	Topic22	Topic22
healthcare-related	Topic14	Topic14	Topic14	Topic14	Topic14
corporations	Topic6	Topic19	Topic12	Topic23	Topic1
non-governmental organizations (NGO)	Topic6	Topic19	Topic2	Topic7	Topic9

**Fig 9:**
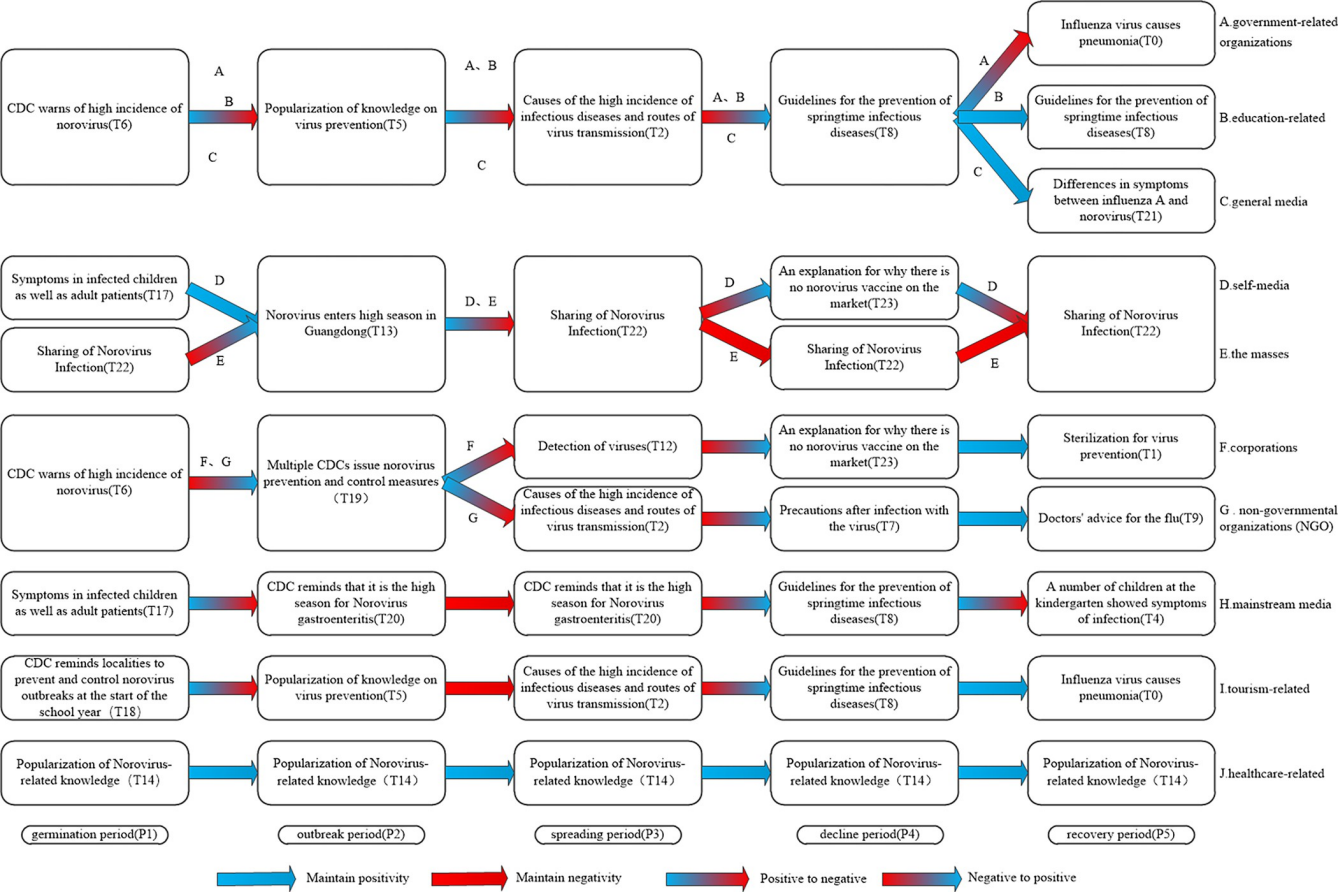
The concern and emotion evolution model of each stakeholder.

From the horizontal perspective, the sentiment values in each stage of the life cycle of public opinion evolution are calculated to obtain the concerns and sentiment evolution patterns of each stakeholder, and those with similar concerns are grouped into one category. The evolution patterns of each stakeholder can be roughly classified into six categories, as shown in Fig 9. Fig 9 represents the topics of greatest concern for each stakeholder at each life cycle stage and the change in sentiment from period to period. In Fig 9, the text in the boxes represents the topics that stakeholders are most concerned about, each column represents each life cycle stage, and each row represents the topics that each type of stakeholder is most concerned about in each period. The arrows represent the emotional changes of each stakeholder in each period, with blue color representing positive emotional tendencies and red color representing negative emotional tendencies. The arrow stays blue to indicate that the affective state remains positive, the arrow stays red to indicate that the affective state remains negative, the arrow changes from blue to red to indicate that the affective state changes from positive to negative, and the arrow changes from red to blue to indicate that the affective state changes from negative to positive.

Firstly, we analyze the theme evolution characteristics of each stakeholder. As depicted in Fig 9, similarities can be observed in the theme evolution process of three stakeholder groups: government-related organizations, education-related and general media; self-media and the general public; and corporations and non-governmental organizations. Specifically, government-related organizations, education-related entities, and general media exhibit complete consistency in their topics of concern during the first four stages of the public opinion life cycle. In the communication process of public health emergencies, the government plays an important role in guiding and channeling public opinion, and plays an important role in public opinion management [27]. After the occurrence of the event, the government-related organizations take the lead in reacting to the event, which is shown in Fig 9: During the germination period, government-related organizations initiate reporting and provide event reminders. As the incident progresses, the government focuses on addressing urgent societal issues, such as virus prevention and the underlying causes of the incident. The media plays the role of information release and dissemination in the evolution of public opinion. After the incident, as a bridge of information dissemination, major media will report on the relevant measures taken by government agencies. In this study, the media are divided into three subcategories: mainstream media, general media, and self-media, and the evolution of the themes of these three subcategories is different, as shown in the following: the general media are more likely to follow up on the initiatives of governmental organizations in the process of public opinion evolution; the mainstream media are more inclined to remind the media of the incident and report on secondary events; and the self-media pay more attention to the themes that are closely related to their own, such as "sharing about norovirus infection". In addition, medical stakeholders prioritize the popularization of virus-related knowledge throughout the evolution of public opinion. Conversely, the general public pays closer attention to the impact of the incident on themselves, focusing on themes related to "sharing of Norovirus infection". As the event progresses, corporations tend to concentrate on topics associated with their own interests, such as "virus detection" and "explanation of the absence of a norovirus vaccine in the market".

Secondly, we analyze the emotional evolution characteristics of each stakeholder. From the perspective of the main entities involved, the emotional evolution process of mainstream media and government-related organizations is similar. Throughout the public opinion life cycle, medical stakeholders consistently maintain a positive emotional state. However, the general public expresses more concerns about the incident and tends to exhibit negative emotions. From the perspective of the public opinion life cycle, during the germination period when the event initially occurs, the amount of information and its impact are relatively small, resulting in fewer people being concerned about the event. As a result, public opinion tends to be positive. In the outbreak period, although negative sentiment increases to some extent, overall, public opinion still tilts towards the positive side. During the spreading period, as the event further intensifies, negative news related to public opinion begins to emerge, leading to a shift in public opinion towards the negative side. However, with the intervention of relevant government departments, they play a role in controlling the further development of public opinion, resulting in a shift back towards a positive sentiment during the decline period. In the recovery period, when the event is essentially over, only a small number of people remain concerned, and the momentum behind the event diminishes. Consequently, public opinion continues to remain positive.

**Fig 10:**
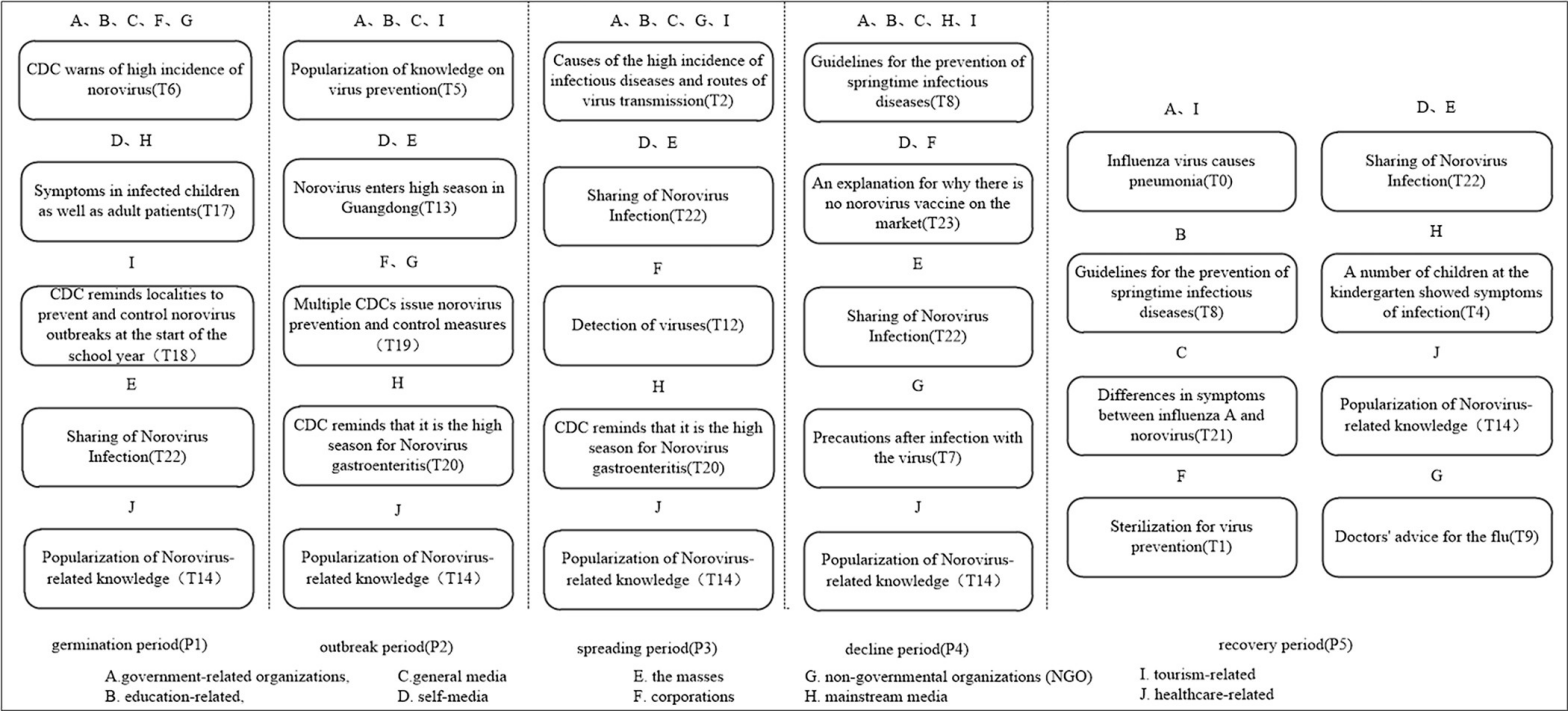
Comparison of concerns of stakeholders in the same life cycle.

Vertically, Fig 10 illustrates the differences in themes of concern among stakeholders within the same lifecycle. In Fig 10, the text in the boxes represents the topics that stakeholders are most concerned about, and the text on the boxes represents the stakeholders who are concerned about that topic. Each column represents a life cycle stage, except for the leveling off period, which consists of two columns.

In the germination stage, "CDC warns of high incidence of norovirus (T6)" is the theme that stakeholders pay more attention to. In the outbreak stage, the focus of stakeholders is shifted to the theme "popularization of knowledge on virus prevention (T5)". During the spreading period, the focus of the population was more on the topic "Causes of the high incidence of infectious diseases and routes of virus transmission (T2)". During the decline period, the focus of the population was not only limited to the current events, and "Guidelines for the prevention of springtime infectious diseases (T8)" became the most popular topic. In the recovery period, the distribution of themes among stakeholders was more dispersed because the public opinion event had basically ended. Overall, in the first four periods, the topic concerns of all types of stakeholders were focused on certain topics, while in the last period the topics of concern of each stakeholder were more dispersed, with a two-stage process of concentration to dispersion of each stakeholder’s concerns.

## Conclusions

This paper examines online public opinion during cross-platform public health emergencies from a platform perspective. By combining stakeholder theory and lifecycle theory, we categorize 10 types of stakeholders and five lifecycle stages of public health emergencies. We employ the LDA+Word2vec model to extract the topics of concern for each stakeholder and utilize the CNN sentiment analysis model to analyze the emotional evolution of each stakeholder. The study investigates the public opinion evolution process for these 10 stakeholder types during each stage of the public opinion lifecycle in public health emergencies. Our findings reveal that the evolution of stakeholder concerns generally follows a two-stage pattern, transitioning from concentration to dispersion. Regarding thematic focus, each stakeholder’s interests are closely intertwined with their respective social domains. For instance, the government assumes a guiding and channeling role throughout the entire public opinion event, starting with initial reminders and warnings, followed by discussions on prevention and the causes of the virus. The government’s involvement plays a pivotal role. On the other hand, medical stakeholders prioritize the dissemination of virus-related knowledge. In terms of emotions, the general public’s sentiment undergoes a three-stage transition from positive to negative and then back to positive. During the early stage of public opinion, a positive sentiment prevails. As the outbreak intensifies, negative emotions among the public increase. The spreading period witnesses the peak of negative emotions. However, with the intervention of government authorities, negative emotions are alleviated during the decline and recovery period.

The findings of this paper will assist relevant government authorities in gaining a more comprehensive understanding of the thematic concerns and emotional evolution process among various stakeholders during public health emergencies. This understanding can enable them to promptly implement appropriate measures to mitigate the losses incurred by such emergencies. It should be noted that this study solely analyzed textual data from public opinion events, thus possessing certain limitations. In future research, a multimodal perspective will be adopted to comprehensively analyze multimodal data, including text, images, videos, and other forms, with the aim of obtaining a more precise understanding of the patterns governing public opinion evolution.

## Supporting information

S1 File Supporting data. (ZIP)

S2 File Sharing code. (ZIP)
